# Prostate cancer stem cells: the role of androgen and estrogen receptors

**DOI:** 10.18632/oncotarget.6220

**Published:** 2015-10-24

**Authors:** Erika Di Zazzo, Giovanni Galasso, Pia Giovannelli, Marzia Di Donato, Annalisa Di Santi, Gustavo Cernera, Valentina Rossi, Ciro Abbondanza, Bruno Moncharmont, Antonio Agostino Sinisi, Gabriella Castoria, Antimo Migliaccio

**Affiliations:** ^1^ Department of Biochemistry, Biophysics and General Pathology, II University of Naples, Naples, Italy; ^2^ Department of Medicine, University of Molise, Campobasso, Italy; ^3^ Endocrinology Section, Department of Cardio-Thoracic and Respiratory Diseases, II University of Naples, Naples, Italy

**Keywords:** prostate cancer, androgen receptor, estradiol receptors, GPR30, stem cells

## Abstract

Prostate cancer is one of the most commonly diagnosed cancers in men, and androgen deprivation therapy still represents the primary treatment for prostate cancer patients. This approach, however, frequently fails and patients develop castration-resistant prostate cancer, which is almost untreatable.

Cancer cells are characterized by a hierarchical organization, and stem/progenitor cells are endowed with tumor-initiating activity. Accumulating evidence indicates that prostate cancer stem cells lack the androgen receptor and are, indeed, resistant to androgen deprivation therapy. In contrast, these cells express classical (α and/or β) and novel (GPR30) estrogen receptors, which may represent new putative targets in prostate cancer treatment.

In the present review, we discuss the still-debated mechanisms, both genomic and non-genomic, by which androgen and estradiol receptors (classical and novel) mediate the hormonal control of prostate cell stemness, transformation, and the continued growth of prostate cancer. Recent preclinical and clinical findings obtained using new androgen receptor antagonists, anti-estrogens, or compounds such as enhancers of androgen receptor degradation and peptides inhibiting non-genomic androgen functions are also presented. These new drugs will likely lead to significant advances in prostate cancer therapy.

## INTRODUCTION

Prostate cancer (PC) represents the most common type of cancer among male individuals and is the second leading cause of cancer death in men. In most cases, PC has a slow and symptom-free growth, whereas in the remaining cases it is more aggressive. Current treatments for clinically localized or advanced PC include radical prostatectomy, androgen deprivation therapy (ADT), radiation therapy, brachytherapy and cryotherapy. However, the efficacy of these therapies still remains unsatisfactory [1]. New therapeutic approaches are, therefore, needed to efficiently hinder PC progression and metastasis.

Since the first observation on the role of androgens in PC progression [2] and the subsequent cloning of the human androgen receptor (AR) [3], it has been widely recognized that the androgen/AR axis controls the growth and development of prostate tissue as well as PC progression. Molecular studies have extensively analyzed the mechanism of AR action in PC, thus allowing the generation of new drugs that inhibit the androgen/AR axis in PC. Among them, ADT still represents the major therapeutic option for advanced PC [4]. Although initially effective in blocking tumor growth, this approach frequently fails and the disease progresses to castration-resistant PC (CRPC). A large number of preclinical and clinical studies are aimed at optimizing the timing of ADT and investigating the molecular basis for PC progression and hormone resistance.

Stem cells (SCs) are present in normal tissues and have the ability to self-renew and differentiate into tissue-specific progeny [5, 6]. Cancer may arise from cells that have the capacity to initiate tumor growth. These cancer-initiating cells, or cancer stem cells (CSCs), may not necessarily be SCs in normal tissues, but may be progenitor cells susceptible to malignant transformation [7]. A particular subset of stem-like cells, defined as cancer-repopulating cells, may constitute a reservoir of self-sustaining cells that self-renew and maintain the tumor. They preferentially reside in specific hypoxic microenvironment-niches inside tumors, often escape from therapies, and are capable of metastatic spreading. A number of reports described the isolation of progenitor cells/SCs from prostate tissues and cell lines, supporting the CSC theory [7-9]. PC cells are highly organized, and only a subset of these cells is endowed with tumor-initiating and long-term tumor-propagating activity, further supporting the CSC theory. In fact, these tumor-initiating cells exhibit some phenotypic and functional features typical of normal prostate SCs and are involved in tumor initiation, metastatic spreading and even drug resistance of PCs [10]. Thus, prostate CSCs may partly account for PC development and progression as well as the refractory state of PC to currently available therapies.

Prostate SCs and CSCs exhibit a robust ability to self-renew in the absence of appreciable amounts of AR [11-13]. Their number significantly increases after bicalutamide (Casodex) treatment or AR-siRNA of various PC cells [14], suggesting that chemical inhibition or absence of AR enhances the self-renewal ability of CSCs. Estrogens also play a role in PC progression. Despite the absence of AR, prostate SCs harbor classical (α and β) and novel (GPR30) estradiol receptors (ERs), which are involved in PC initiation and progression [12, 15].

Here we discuss the putative role of these receptors in prostate SCs and CSCs. New therapeutic strategies based on the use of novel compounds (antagonists, small peptides interfering in non-genomic AR action, enhancers of AR degradation) are also reviewed, since they might provide important advances in PC treatment.

## PROSTATE ORGANIZATION

Prostate gland is made up of basal, luminal, and neuroendocrine cells embedded in a fibro-muscular stroma [16, 17]. Luminal secretory cells, which represent the major epithelial cell population, express both cytokeratin 8/18 and AR. They are, therefore, androgen dependent for growth and survival. Luminal secretory cells are more sensitive to the local microenvironment and more susceptible to genetic and epigenetic modifications upon exposure to oxidative stress and DNA damage. Basal epithelial cells are aligned between the basement membrane and luminal cells, and express p63 and cytokeratin 5. Because of the absence of AR, they are androgen independent. Neuroendocrine cells, identified by the expression of chromogranin A and synaptophysin, have unknown functions.

Cell lineage is a key factor in PC development, since the majority of PCs (95-99%) arise from the luminal cell lineage, with only 1-5% deriving from the neuroendocrine cell lineage. Basal cell-derived PCs have not been reported to date [1]. A well-accepted hypothesis is that individual cancers originate from SCs or dividing progenitor cells. A rare population of SCs exists within the human prostate gland, together with another rare population of intermediate cells that are likely progenitor cells and express cytokeratin 5, cytokeratin 8/18 and prostate stem cell antigen [15].

PC may originate from SCs that become CSCs as a consequence of accumulating mutations. Thus, prostate CSCs might arise from SCs or progenitor cells or differentiated cancer cells undergoing aberrations as a consequence of genetic mutations or changes in tumor microenvironment [18]. CSCs exhibit the unlimited ability to self-renew [10], have a mesenchyme-like phenotype and express specific surface antigens (e.g., CD133 and CD44), useful for their isolation [19]. Relevant to the findings discussed here is the observation that cytokeratin 5^+^ stem/progenitor or intermediate cells increase after ADT or in CRPC [14, 20-23]. Nonetheless, the identification of prostate CSCs remains controversial, and their contribution to PC progression is still a matter of discussion.

## AR IN PROSTATE CANCER

The human AR gene encodes for a protein with a mass of 110 KDa that consists of an N-terminal domain, a DNA-binding domain and a ligand-binding domain. The ‘hinge region’, which separates the ligand-binding domain from the DNA-binding domain, is made up of a short amino acid sequence that contains part of a bipartite ligand-dependent nuclear localization signal responsible for AR nuclear translocation [24].

**Figure 1:**
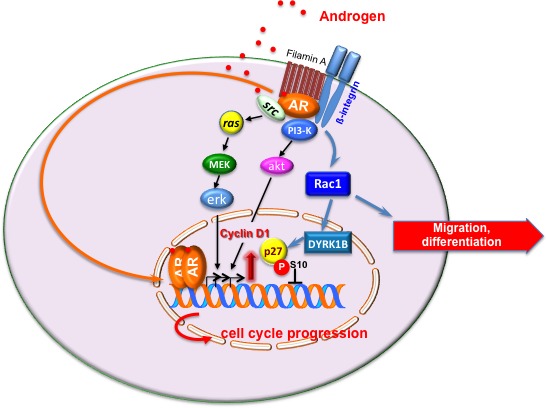
Model of androgen action in target cells The transcriptional and non-transcriptional models of androgen action in target cells are depicted. Upon ligand binding, cytoplasmic AR dimerizes and translocates into nuclei of target cells, where it activates gene transcription [1, 4, 24, 80]. In the extra-nuclear compartment of target cells, ligand-bound AR recruits and activates various signaling effectors, including Src, PI3-K, β1-integrin and filamin A. Stimulation of target cells with androgens triggers cell cycle progression through AR/Src/PI3-K complex assembly [28, 31]. Androgens also induce the assembly of AR/filamin A/β1-integrin complex. This complex activates Rac, thereby inducing motility or differentiation in target cells [29, 30]. Under certain conditions, the androgen-triggered AR/filamin A complex activates the Rac/dual-specificity tyrosine-phosphorylation regulated kinase 1B (DYRK1B) pathway, leading to p27 Ser10 phosphorylation and p27 stabilization. Reversible quiescence of target cells follows [37]. By this mechanism, androgens might offset the growth-promoting functions driven by oncogenic Ras [37] or growth factors (unpublished results).

**Figure 2:**
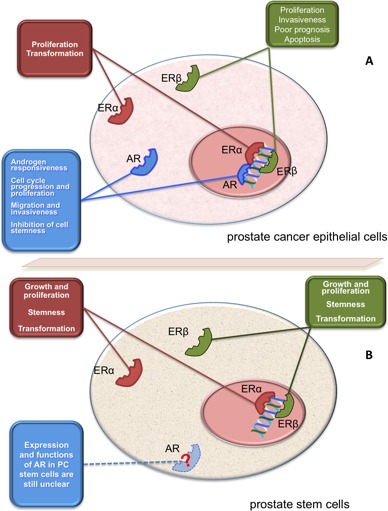
Function of AR and ERs in PC epithelial cells and prostate SCs Panel A illustrates the putative role of AR and ERs (α or β) in epithelial PC cells. Depending on experimental setting, these receptors mediate the indicated biological responses in PC cells [27]. Panel B illustrates the putative role of AR and ERs (α or β) in prostate SCs or CSCs. With few exceptions [103], AR is almost undetectable in prostate and PC SCs [8, 11-15, 93-95]. Prostaspheres derived from primary human prostate epithelial cells express ERs (α or β1) that activate transcriptional and non-transcriptional mechanisms, thus sustaining growth, transformation and stemness [12, 15, 102]. PC epithelial cells and prostaspheres derived from primary human prostate epithelial cells also express the novel ER, GPR30 [12, 15, 85, 86].

AR controls the growth of prostate gland and is a hallmark of PC, since it is expressed throughout the various stages of the disease [25]. Classically, the androgen-bound AR enters cell nuclei, binds discrete nucleotide sequences in DNA and stimulates gene transcription [24] (Figure 1). Additionally, ligand-bound AR acts in target cells at non-transcriptional level [26]. Thus, ligand-bound AR triggers rapid and transient activation of Src tyrosine kinase, increases the active form of small-GTP binding proteins (Ras and Rac 1) and stimulates the activity of mitogen-activated protein kinases as well as focal adhesion kinase in target cells [27]. Androgens activate these signal transduction pathways *via* the direct interaction of AR with Src-SH3 domain [28] or filamin A [29, 30]. Androgens also trigger PI3-K/Akt pathway activation, thereby increasing cell proliferation [31] (Figure 1). To what extent the activation of extra-nuclear pathways by androgens contributes to prostate transformation and PC progression is still debated. Some years ago, we synthesized the S1 peptide that mimics the AR region involved in its interaction with Src-SH3 domain, thereby inhibiting the growth of PC cells cultured *in vitro* or xenografted in mouse [32]. Recently, it has been reported that PC invasion is stimulated by a rapid and sustained increase in Src activity, mediated by non-genomic AR action [33]. These findings indicate that non-genomic pathways engaged by AR impinge on PC proliferation and invasiveness. Figure 2A summarizes the biological responses elicited by AR in epithelial PC cells.

Additionally, our recent results in primary mouse embryo fibroblasts and NIH3T3 as well as human fibrosarcoma HT1080 cells provide new clues about the role of AR. While androgens do not induce significant cell growth, they do enhance cell motility in these cells by stimulating AR interaction with filamin A [29]. We recently obtained similar findings in primary cultures of fibroblasts from PC specimens (unpublished data).

Filamin A and its proteolytic fragments directly interact with the 622-670 sequence of AR, thereby modulating the nuclear import and transcriptional activity of AR or the androgen responsiveness of LNCaP cells [34-36]. We recently observed that AR interacts and co-localizes with full-length filamin A in the extra-nuclear compartment of NIH3T3 and HT1080 cells, as well as in neuronal PC12 cells. The androgen-triggered AR/filamin A bipartite complex is required for motility [29, 37] or neuritogenesis [30] of these cells. These results support the conclusion that the androgen-triggered AR/filamin A complex commands motility and adhesion when poised at cytoplasm of target cells. Of interest, high levels of cytoplasmic filamin A have been detected in metastatic PC specimens [38], and a significant increase in filamin A-rich actin structures localized at the cell periphery has been revealed by proteomic analysis of aggressive PC cells [39]. These findings make the extranuclear AR/filamin A complex a good candidate for invasiveness-initiating activity and a new therapeutic target in PC spreading.

With the aim of discovering new drugs inhibiting AR-dependent motility, we synthesized an AC-stapled (A628S5, K632S5)-amide peptide from the AR 628-646 sequence responsible for its interaction with filamin A, in which Ala628 and Lys632 residues were each replaced with an olefinic amino acid that allows them to be cross-linked. The peptide is thus ‘locked’ into its bioactive alpha-helical fold through the insertion of hydrocarbon staples. This modification increases affinity for targets as well as cell permeability, and decreases degradation of the stapled peptide, as compared to its unstapled counterpart [37]. The stapled peptide specifically displaces the androgen-triggered interaction of AR with filamin A, thus inhibiting motility and differentiation triggered by androgens in mesenchymal as well as neuronal cells. Notably, the peptide leaves unaltered AR-mediated gene transcription, and only acts in AR-expressing cells [30, 37]. These properties make the peptide a good candidate for further translational studies in view of therapeutic applications.

Whatever the mechanism (transcriptional *versus* non-transcriptional), AR is constantly expressed in PC and its decrease reduces both androgen-independent and CRPC types [4]. By enhancing the association of AR with the Mdm-2 ubiquitin-ligase, the new compound ASC-J9 promotes AR degradation, reduces androgen binding to AR and the consequent AR nuclear translocation. These inhibitory functions lead to suppression of AR-mediated cell growth [40]. Because of these properties, the compound ASC-J9 has recently emerged as a powerful new approach in PC treatment.

CRPC is commonly attributed to the reactivation of AR transcriptional function (likely due to AR gene amplification or mutation) or AR activation by alternative signaling effectors [1]. Analysis of AR aberrancies (amplification, mutations, splice variants) has attracted the attention of many investigators, because of the possibility to specifically target the most common AR variants in PC [4]. AR variants lacking the C-terminal ligand-binding domain are often expressed in CRPC cells and PC clinical samples. These variants are constitutively active, and therefore resistant to traditional ADT. Their expression is even induced by ADT, making them new putative targets for the treatment of CRPC [41].

## ERS IN PROSTATE CANCER

Genetically engineered, xenograft and cell culture models currently available do not accurately represent the molecular and cellular events underlying PC development and progression to CRPC. While the AR status of the most commonly used cell lines is generally known, expression levels of ERα and ERβ vary across different cell lines, and conflicting data have been reported for each cell type [42, 43]. Thus, many PC cell lines are unable to exactly reproduce the cross talk between ER (α or β) and AR occurring at extra-nuclear level in LNCaP cells [28], or the epidermal growth factor (EGF)-induced intersection between ERβ/AR/EGF receptor in LNCaP cells [44]. Again, some PC cells are unable to replicate the interplay between ERα and ERβ taking place at gene transcription level [45]. These findings partly explain the conflicting data thus far reported on estrogen action in PC cells. Again, the most routinely used PC cells do not represent human tissue, since ERα expression is predominantly confined to stroma of human prostate, while luminal epithelial cells express ERβ and AR, and basal epithelial cells only express ERβ [46, 47]. Therefore, PC cells do not reproduce the stromal-epithelial interactions known to be important in cancer development and progression [48]. Additional findings have, however, contributed to our scant knowledge concerning the role of estrogens and ERs in the etiology and progression of PC. Estrogens, in combination with androgens, induce prostate transformation and PC progression [12]. Chronically elevated estrogen levels are correlated with increased PC risk, and ER expression changes during PC progression. Testosterone, in combination with estradiol, induces prostatic hyperplasia and dysplasia in mice as well as PC in rats [49]. Clinical and epidemiologic studies have shown that European men have a low risk of developing PC, as compared with African-American men. PC incidence is still lower in Japanese men [50-52]. These findings warrant some further comments. Since testosterone levels are similar in the three groups [51] and estrogen levels are higher in African-American men than in Caucasian men [53, 54], a direct correlation between serum estradiol levels and PC risk was hypothesized. However, no such correlation has been definitively proved [55, 56]. It was previously reported that excessive exposure to estrogens during development might contribute to the high incidence of benign prostatic hyperplasia and PC, and maternal exposure to diethylstilbestrol during pregnancy enhances prostatic metaplasia in male offspring [57]. Based on these and other findings, it was argued that prenatal exposure to diethylstilbestrol increases PC risk [58]. Studies in rodent models confirmed that exposure to diethylstilbestrol or pharmacologic levels of estradiol *in utero* enhance susceptibility to PC [59-62]. Use of diethylstilbestrol during pregnancy was finally stopped in the 1970s. Nevertheless, other endocrine disruptors with estrogenic activity are implicated in PC risk. Bisphenol A, for instance, influences prostate transformation and proliferation in rodent models and human prostate cell lines. It is also involved in progression of PC expressing AR variants [15]. Further evidence supports a role for estradiol in PC. PC incidence increases during aging, when serum testosterone levels decline but estrogen levels remain constant, suggesting that the estradiol/testosterone ratio, rather than serum levels of each steroid, is critical in PC development [63]. These findings raise many concerns. Firstly, how and when do estradiol levels rise in aging males? Multiple estrogen sources may account for this increase. Adipose tissue represents a *de novo* estrogen source [64] and aging males exhibit an increase in female-type fat deposition that might promote PC. Again, estrogen levels might locally increase in PC [65]. Secondly, many labs have detected a significant decrease in bioavailable testosterone and estrogen levels over the male lifespan [66-72]. The use of different techniques in estradiol assays could explain the conflicting findings on bioavailable steroid levels reported during aging. The application of advanced technologies allowing an accurate assay of ultra-low and intra-tissue estradiol levels [73] should be pursued to better address this issue. Further interest in the role of estradiol in PC has been raised by clinical data showing beneficial effects of transdermal estrogen therapy in CRPC patients [74]. In conclusion, depending on cell type (stem, developing or transformed cells) and PC stage, physiological estrogens or xeno-estrogens might exert quite divergent effects.

ERs, α or β, mediate estrogen effects in target cells [75]. Human prostate expresses both ER isoforms and is targeted by estrogens. ERα is predominantly expressed in stroma, while ERβ is detectable in epithelium of prostate gland [49, 76, 77, 78]. Once bound to estradiol, these receptors influence cell responses by a complex interface between signaling cascade activation and control of gene expression [79]. Genomic effects usually occur *via* ligand-dependent binding of ER (α or β) to target gene promoters [80]. Estradiol action in malignant prostate tissue and ER-expressing prostate epithelial cells involves, for instance, the nuclear lysine histone protein methyltransferase retinoblastoma-interacting zinc-finger protein 1 (RIZ 1), a downstream effector of estrogen in estradiol-target tissues. In prostate epithelial cells, RIZ 1 promotes prostate cell maturation and hormone responsiveness. The RIZ 2 variant form lacks the histone methyltransferase domain, and likely induces hormone independence and malignancy of prostate tissue [81].

ERs also induce rapid, non-genomic effects independently of RNA or protein synthesis. Thus, ERs localized in the extra-nuclear compartment of target cells activate signal transduction pathways involving Src, MAPKs, adenylyl cyclase and PI3-K, or mediate a rapid increase in intracellular calcium levels [27]. We also reported that rapid estradiol action mediated by ERβ drives cell cycle progression and proliferation in LNCaP cells [28]. Similarly, extra-nuclear action mediated by ERβ positively affects proliferation and motility of PC cells challenged with EGF, suggesting that ERβ intersects growth factors and their cognate receptors in PC cells [44]. The finding that isoforms 2 and 5 of ERβ positively correlate with invasiveness and poor prognosis in PC patients [82] is consistent with data obtained in our lab. Nevertheless, it has also been shown that ERβ activation induces apoptosis in prostatic stromal, luminal and castration-resistant basal epithelial cells of estrogen-deficient aromatase knockout mice. ERβ activation also causes apoptosis in Gleason grade 7 xenografted tissues as well as in androgen-independent PC3 and DU145 cell lines [83]. Additionally, ERα knockout mice do not develop high-grade prostate intraepithelial neoplasia or PC [65], suggesting that prostate proliferation and transformation induced by estrogens is related to ERα, rather than ERβ. Figure 2A depicts the action mediated by ERs (α or β) in epithelial PC cells.

The conflicting evidence on the role of ERs (α or β) collected so far in different experimental conditions (i.e., immune-depressed mouse and xenograft models, normal or transformed cultured cells, PC biopsies) suggests that further analysis of ERα or β action should be carried out to gain valuable findings in relation to PC. The scenario seems to be even more complex because of the putative role of the novel ER, GPR30 in PC progression and androgen resistance. GPR30 contributes to physiological effects elicited by estrogens in target cells. In addition to mediating some of the rapid signaling events on estradiol stimulation (e.g., calcium mobilization and kinase activation), GPR30 also regulates gene transcription [84]. A role for GPR30 in PC has been also reported. Activation of GPR30 by the non-estrogenic ligand G-1 inhibits the growth of androgen-dependent and androgen-independent PC cultured cells and PC-3 xenografts *in vivo* [85]. Again, GPR30 expression is significantly higher in castration-resistant PC than in androgen-sensitive PC clinical specimens. Additionally, G-1 ligand inhibits the growth of castration-resistant but not androgen-sensitive PC [86]. Thus, GPR30 may represent a new therapeutic target in CRPC. We now appreciate, however, that prostate cells are sensitive to estradiol, substantiating the use of specific ER modulators (SERMs), which may be ERα or ERβ agonists or GPR30 ligands in PC pharmacotherapy. Raloxifene, a SERM selectively activating ERβ and with prevalent antagonism for ERα, induces apoptosis in both androgen-dependent and -independent PC cells. It also inhibits the growth of human PC xenografted cells [87-91]. Overall, clinical findings have recently confirmed some beneficial effects of the anti-estrogen toremifene in patients with prostate intraepithelial neoplasia [92].

## AR IN PROSTATE SCS AND CSCS

Prostate SCs or progenitor cells prevalently reside in the basal, but not in the luminal layer of human prostate epithelium. Since basal cells are AR negative, while luminal cells express high levels of AR, it was previously hypothesized that prostate SCs express undetectable levels of AR [11]. This hypothesis was subsequently confirmed by experimental findings, and many reports indicated that prostate SCs or CSCs exhibit almost undetectable levels of AR, or even lack the receptor [8, 12-15, 93-95] (see also Table I).

**Table I:** Expression of sex steroid receptors in prostate SCs and CSCs

RECEPTOR	EXPRESSION	EXPERIMENTAL EVIDENCE	REFERENCES
AR	undetectable	q-RT-PCR Western blot immunohistochemistry immunofluorescence	[8, 11-15; 93-95]
	detectable	dual-variable flow cytometry	[103]
ERα	detectable	q-RT-PCR immunohistochemistry immunofluorescence	[12, 15]
ERβ	detectable	q-RT-PCR immunohistochemistry immunofluorescence	[12, 15, 154]

A number of markers (i.e., CD133, CD44, integrin α6, integrin α2β1 and Sca-1) have been used to identify or isolate prostate SCs or CSCs [14, 96, 97]. Basal cells exhibit stem cell properties, as shown by their positivity to Sca-1 in mouse and CD133 in humans. Expression of AR in these cells is almost undetectable [98, 99]. It is now largely accepted that androgen sensitivity and AR expression in prostate tissue are both organized in a hierarchical model in which cytokeratin 5^+^8^−^ basal cells, containing SCs or progenitors, lack AR. In contrast, cytokeratin 5^+^8^+^ intermediate and cytokeratin5^−^8^+^ luminal cells express AR [100, 101]. It should be noted, however, that stem cells may also be present in the luminal cells of regenerating prostate [102]. Again, conflicting data have been reported on AR expression in prostate CSCs. Many years ago it was reported that CD133^+^ cells lack AR. In contrast, subsequent studies reported that CD133^+^ tumor-initiating cells express AR, as assessed by dual variable flow-cytometry [103]. However, the concept that prostate CSCs do not express AR, and hence do not respond to androgens, has also been corroborated by several indirect findings. PC cells exhibiting low AR-mediated gene transcription acquire the ability to self-renew upon castration [13]. Castration increases the amount of prostate CSCs [14], and AR-negative PC cells show a very high proliferative rate, together with stem/progenitor cell characteristics [97]. In fact, spheroid structures, containing SCs or progenitor cells, more frequently develop in AR-negative than in AR-positive PC-derived cells [104]. Depleting these latter cells of AR leads to an increase in the number of spheres and their CSC or progenitor cell content [14]. In sum, AR differentially regulates the functions of PC cells or prostate CSCs, since its expression seems to confer hormone responsiveness to PC epithelial cells, while its absence or chemical inhibition might enable acquisition of cell stemness (Figure 2B).

The finding that prostate SCs and CSCs do not express AR calls for further comments. The use of cell markers to isolate SCs and prostate CSCs may generate a mixed cell population, significantly complicating the identification of pure AR-negative or -positive cells. Additionally, several normal or transformed cell types express low levels of AR, making the receptor unable to dimerize and translocate into the cell nucleus. These cells have, in most cases, been considered androgen insensitive because of their very low AR expression level, and hence the absence of AR-mediated gene transcription. Nonetheless, they still respond to androgens with activation of signaling effectors [105]. Moreover, the findings so far reported on AR expression in prostate SCs or CSCs do not exclude that membrane AR might be expressed in these cells. Palmitoylation, for instance, regulates AR localization at cell membrane [106]. AR also interacts with caveolin 1, thereby localizing in lipid rafts within the membrane [107]. Thus, further analysis using new impeded ligands, biochemical approaches to isolate rafts and visualize AR by imaging techniques as well as quantitative immunoreactive plate assay [108-110] should be performed in an attempt to reach any definitive conclusion. In sum, the study of AR expression and function in prostate SCs and CSCs warrants further investigation. Table I reports the data currently available on AR expression in prostate CSCs.

Various signaling pathways are involved in prostate CSC functions. PI3-K/Akt pathway activation sustains survival and proliferation of PC progenitor cells. Again, Ras/MAPK and Stat3 signaling both sustain self-renewal of prostate CSCs [111]. Experimental findings collected in the last decade indicate that AR mediates activation of PI3-K and Ras-dependent pathways upon androgen stimulation of target cells. Specifically, ligand-bound AR increases PI3-K activity through a non-transcriptional mechanism in cytoplasm, while it antagonizes PI3-K activity *via* a transcriptional mechanism in nuclei. Rapid activation of PI3-K/Akt signaling by androgens impinges on proliferation and differentiation of various cell types [31]. A direct interaction of AR with the p85α regulatory subunit of PI3-K has been observed upon re-expression of AR in PC3 cells or androgen stimulation of normal epithelial cells [112]. Conversely, down-regulation of PI3-K/Akt pathway by nuclear AR activity has been observed in prostate PTEN-deficient mouse, human PC and N-methyl-N-nitrosourea prostate tumor-induced rat model. In this latter setting, the staining of P-Akt increases as the amount of nuclear AR decreases [113, 114]. Therefore, it can be argued that when AR cytoplasmic functions are expanded, the receptor activates PI3-K pathway, thus enabling PC cell proliferation and stemness. In contrast, when AR nuclear functions are fostered, the receptor inhibits PI3-K/Akt pathway, thereby limiting PC cell proliferation and stemness. In this scenario, AR intracellular localization might command cell stemness in PC. It has been consistently shown that steroid receptor localization orchestrates the biological outcome in target cells [29, 30, 31, 115-119].

Most PC-related deaths are due to metastatic spreading, and human metastasis-initiating cells (MICs) have been found within sub-populations of CSCs that often express the features of epithelial-mesenchymal transition (EMT). These cells migrate and are putative candidates for metastasis-initiating activities [120]. However, the role of the androgen/AR axis in PC metastatic spreading is still debated. Results from orthotopically recombining stromal WPMY1 cells with epithelial PC3 cells in mice have shown that restoring AR in PC3 cells or silencing AR in WPMY1 cells inhibits PC metastasis. Knockdown of AR in CWR22rv1 PC cells also enhances invasion *in vitro* and *in vivo*. Re-expression of AR in PC3 cells decreases invasiveness in bone lesion assay and *in vivo* [121]. By inhibiting the AR axis through the release of cytokine CCL5, infiltrating bone marrow mesenchymal stem cells increase prostate CSC population and PC metastatic properties [122]. Again, ADT induces EMT in normal mouse prostate tissue as well as in human LuCaP35 prostate tumor explants [123], and the anti-androgens bicalutamide and enzalutamide both increase invasiveness in different PC cell lines (LNCaP, C81, C4-2, and CWR22Rv1) and CWR22Rv1 xenografted mouse model [124]. In conclusion, findings in PC cell lines *in vitro* or in xenograft models suggest that the androgen/AR axis plays a suppressor role in PC invasiveness. In contrast, much evidence implicates AR signaling in PC metastatic spreading at early and late stages. AR might promote pro-metastatic events through derangement of chemokine/chemokine receptor functions [125]. AR controls the expression of chemokine receptors as well as their ligands [126, 127]. CXCR4 is overexpressed in clinical specimens from both primary and metastatic PC, as compared with benign controls [128]. CXCR4-expressing PC cells migrate towards a stromal-derived factor-1 gradient, provided that the androgen/AR axis is intact [127]. However, other AR-related networks are involved in metastatic events. AR coregulators enhance invasiveness *in vitro* and *in vivo*. Depletion of FOXA1 fosters PC metastasis and increases patient mortality [129]. Deregulation of the chromatin remodeling SWI/SNF complex is correlated with AR-driven metastatic events and the SWI/SNF subunit, BAF57 is significantly elevated in clinical specimens of metastatic PC [130]. By modulating the expression of Slug transcription factor, cyclin D1b promotes AR-dependent metastatic events in various PC cells *in vitro* and *in vivo*. Clinical findings from CRPC specimens revealed a significant positive correlation between cyclin D1b and Slug expression, indicating that the cyclin D1b/AR/Slug axis is preserved in human PC [131]. Fusion genes and transcriptional effectors are also involved in PC metastasis. Chromosomal fusion between the AR-responsive TMPRSS2 gene and the erythroblast transformation-specific (ETS) gene results in a fusion protein, TMPRSS2:ETS, which is associated with metastatic PC [132]. SOX9, a member of the SOX transcription factor family, cooperates with TMPRSS:ETS in inducing metastatic events in PC cells or heterozygous phosphatase and tensin homolog (PTEN)-deficient mouse. PC patients expressing TMPRSS:ETS often exhibit high levels of SOX9, a signature that is further increased in metastatic PC [133]. In conclusion, clinical findings support a role for AR-driven pathways in metastatic events. Nevertheless, metastatic PC often acquires the ability to produce its own androgens, and hence continuously reactivates AR, despite ADT. Thus, it is not unexpected that in some circumstances ADT increases PC invasiveness. Abiraterone acetate, an inhibitor of androgen synthesis, has been approved for the treatment of metastatic PC in patients exhibiting disease progression in spite of ADT [134, 135].

Altered function of extra-nuclear AR-associated signaling effectors or scaffolds might also be associated with PC metastatic events. We recently observed that AR-driven non genomic events trigger invasiveness of PC cells when they transit from hormone-dependent to hormone-independent status (unpublished data), making the extra-nuclear AR the culprit for invasiveness-initiating activity and maintenance of metastatic phenotype. A role for non-genomic AR functions was observed with regard to motility and invasiveness of various AR-expressing cancer cell types (e.g., fibroblasts and PC-associated fibroblasts, fibrosarcoma, breast, prostate, colon and pancreas tumor cells) [29, 136 and unpublished data]. Of note, PC-associated fibroblasts enhance prostate transformation, promote tumor progression and often represent a stromal ‘niche’ that sustains the function of CSCs [137-139]. Although immunohistochemistry analysis has shown that AR expression is very high in non-malignant stroma, as compared with PC stroma [140-143], different approaches (immunofluorescence, qPCR and Western blot) have revealed appreciable levels of AR in fibroblasts from PC specimens [144]. Similarly to results obtained in primary mouse embryo fibroblasts or NIH3T3 and HT1080 cells [29, 31, 37, 136], we have observed that primary PC-associated fibroblasts express AR, which mediates cell motility but leaves unaffected cell proliferation upon androgen stimulation (unpublished results). In sum, it can be argued that AR directs fibroblasts towards epithelial PC cells upon a local increase in androgen levels. At this stage, AR might promote PC metastasis by changing the tumor microenvironment composition or inducing the release of stromal growth factors [143, 145]. Finally, a malignant cross talk might occur between stromal and epithelial PC cells, enabling the gain of metastatic properties and stemness traits. Therefore, the functions of stromal AR and epithelial-stroma interactions should be more extensively explored in the light of their role in prostate CSC behavior and PC therapeutic approaches. In most cases, current therapies for PC only target the functions of epithelial AR and often produce unwanted effects. In addition to enhancing PC metastatic spreading and SC population [124, 146], ADT, for instance, enables the proliferation of AR-expressing fibroblasts and likely the stromal collagen content [37]. Consequently, ADT might restrain the growth of PC cells by building a stromal-derived niche, while facilitating PC escape from AR inhibition. These findings further highlight the complexity in making the most appropriate therapeutic choice in PC treatment.

## ERS IN PROSTATE SCS AND CSCS

As discussed above, the role of estrogens in the etiology and progression of PC still remains unclear. A model for isolating SCs from mixed epithelial cell cultures utilizes a 3-D cell culture system, wherein only SCs survive and proliferate, forming spheroid structures of SCs or progenitors, also named prostaspheres. This system, initially used to isolate neural stem cells, has been successively extended to SCs from different tissues, including prostate [12, 147-150]. The prostasphere assay allows the isolation and expansion of prostate SCs or progenitors *in vitro*, thereby enabling analysis of growth and differentiation. Using this assay, it was shown that Wnt/β-catenin signaling activation increases the prostasphere size and self-renewal ability of PC cells [151]. Therefore, this approach is now commonly used to study the functions of prostate SCs.

In addition to exhibiting membrane-associated prostate SC markers, such as CD117, Trop2, CD49f and ABCG2, prostaspheres derived from primary cultures of normal human prostate epithelial cells express almost undetectable levels of AR mRNA and are AR negative at protein level. In contrast, these cells express ERs (α or β1 or GPR30), in combination with high levels of retinoic acid receptor and retinoid X receptor. ER mRNA expression levels are significantly higher in normal progenitor cells than in LNCaP cells, with 6-fold higher ERβ1, 15-fold higher GPR30, and 125-fold higher ERα expression. These findings identify prostaspheres as potential targets for estradiol and retinoids [12, 15]. Additionally, no significant differences in expression levels of ER (α or β) were detected by qPCR in SCs from primary normal or malignant prostate cells [152]. Table I reports the data currently available on expression of ERs (α or β) in prostate SCs and CSCs.

Normal prostaspheres derived from primary cultures of human prostate epithelial cells proliferate and grow upon stimulation with 1 nM estradiol [12]. Findings obtained using side-population FACS analysis of primary prostate epithelial cell cultures showed a dose-dependent increase in SC number after 4 days of culture in 10-1000 nM estradiol [15]. Thus, estrogens target prostate SCs or progenitors. These hormones might enhance PC risk by increasing the number of SCs or progenitors and expanding the number of cells undergoing transformation (Figure 2B). However, estrogens might control prostate transformation by inducing epigenetic modifications in prostate SCs or progenitors. Estrogens directly induce transformation in prostaspheres derived from primary cultures of human prostate epithelial cells through activation of Akt and Erk. These non genomic events might sustain cell stemness and transformation. Simultaneously, these prostaspheres also respond to estradiol by increasing gene transcription [153]. Although intriguing, these results do not provide insight into the mechanisms by which estrogen-activated cytoplasmic or nuclear pathways impinge on cell stemness and transformation. Nevertheless, these data might account for some beneficial effects exerted by antiestrogens in PC patients.

Findings obtained in prostaspheres derived from primary cultures of human prostate epithelial cells raise another important question. Since ERα and β are both expressed in prostaspheres, it might be argued that each receptor isoform mediates specific cellular responses upon estradiol binding. ERα or β might be specifically involved in non-genomic or genomic effects elicited by estradiol, thus contributing in a different way to hormone action. Despite intense investigations, we still have limited possibilities for using ligands that specifically bind ERα or β or even synthetic ligands that specifically affect genomic or non-genomic actions of steroids. Findings obtained using the synthetic ligand 8β-VE2, a specific ERβ agonist, showed that it induces apoptosis in both castration-resistant prostate CSCs [154] and a stem/progenitor CD133^+^ subpopulation of the BPH-1 cell line [83]. Thus, specific activation of ERβ by a synthetic agonist might exert beneficial effects in CRPC, making the scenario even more complex.

## CONCLUDING REMARKS

PC represents the major cause of cancer-related death among the male population of Western society, and ADT is still used as the first line in PC treatment. However, ADT frequently fails or even induces paradoxical effects. The androgen/AR axis in PC and prostate CSCs has been investigated for several years. Much evidence from preclinical and clinical studies has shown that multiple androgen/AR signaling pathways operate in prostate tissue. Derangement of these pathways contributes to aberrant growth, maintenance of malignancy and PC invasiveness. At this stage, the disease becomes resistant to castration and is almost untreatable. In recent years, many therapeutic tools (e.g., new antagonists, enhancers of AR degradation, small peptides interfering in AR-mediated non-genomic events) have been designed to specifically target the functions of epithelial or stromal AR, or even the extra-nuclear functions of AR. Many of these molecules have been successfully exploited in preclinical or clinical models. Nonetheless, additional events sustain the disease and its escape from therapy. The role of estradiol/ER axis in PC and prostate CSCs is undeniable. Estradiol action in PC is commonly considered AR independent, with the focus on putative opposite effects, adverse or beneficial, exerted by the two ER isoforms, α and β, respectively. The novel ER, GPR30, has recently emerged as a therapeutic target in CRPC. Since PC co-expresses classical ERs and GPR30, a high degree of synergism and antagonism between various ligands and antagonists likely occurs in PC, adding further level of complexity to an already difficult therapeutic approach.

Despite major advances in our understanding of the molecular basis of PC and in the generation of highly sophisticated tools for biological research, the questions addressed here suggest that we have to profoundly re-examine the mechanism of sex steroid receptor action in PC to better exploit new emerging targets and drugs. The power of novel genetically-engineered animal models or high-throughput screening in PC cells and/or biopsies will likely improve the design of new compounds that inhibit sex steroid receptor functions, leaving other useful receptor activities unaltered.

## Abbreviations

ADT: androgen deprivation therapy
AR: androgen receptor
CRPC: castration-resistant prostate cancer
CSC: cancer stem cell
EGF: epidermal growth factor
ERs: estrogen receptors
MAPK: mitogen-activated protein kinase
SC: stem cell
PC: prostate cancer
PI3-K: phosphatidylinositol-3-kinase
